# Serial Exhaustive Extraction Revealed Antimicrobial and Antioxidant Properties of *Platycerium stemaria* (Beauv) Desv

**DOI:** 10.1155/2021/1584141

**Published:** 2021-06-16

**Authors:** Vincent Ngouana, Elisabeth Zeuko'o Menkem, Diane Yimta Youmbi, Lorette Victorine Yimgang, Rufin Marie Kouipou Toghueo, Fabrice Fekam Boyom

**Affiliations:** ^1^Department of Pharmaceutical Sciences, Faculty of Medicine and Pharmaceutical Sciences, University of Dschang, P.O. Box 96, Dschang, Cameroon; ^2^Antimicrobial and Biocontrol Agents Unit, Laboratory for Phytobiochemistry and Medicinal Plants Studies, Department of Biochemistry, Faculty of Science, University of Yaoundé I, P.O. Box 812, Messa, Yaoundé, Cameroon; ^3^Department of Biomedical Sciences, Faculty of Health Sciences, University of Buea, PO Box 63, Buea, Cameroon

## Abstract

Microbial infections are increasing worldwide, and the widespread emergence of antibiotic-resistant pathogens poses a severe threat to public health. Medicinal plants are well-known sources of bioactive ingredients. This study was designed to determine the antimicrobial and antioxidant activities of extracts from *Platycerium stemaria*. The serial exhaustive extraction method using a solvent of increasing polarity from nonpolar (hexane) to polar (water) was designed to prepare crude extracts; liquid-liquid partition was used to fractionate of active extracts. The extracts and fractions were screened for antimicrobial activity on bacteria and yeasts using the microdilution method. The antioxidant activity was done using DPPH and FRAP assays. Out of the sixteen extracts screened, four (*Ps*Hex, *Ps*H2O(H), *Ps*MeOH(EA), and *Ps*MeOH) exhibited potency with minimal inhibitory concentration (MIC) values ranging from 31.25 to 500 *μ*g/mL. Out of the four extracts, two, including *Ps*MeOH and *Ps*MeOH(EA), exhibited DPPH radical scavenging activity with the antiradical power of 8.94 × 10^−5^ and 47.96 × 10^−5^, respectively, and ferric reducing antioxidant power values ranging from 0.34 to 61.53 *μ*g equivalent Vit C/g of extract. The phytochemical screening of the promising crude extracts revealed flavonoids, glycosides, phenols, tannins, terpenoids, saponins, and anthraquinones. This study reports the antimicrobial and antioxidant activities of *P. stemaria* for the first time. The results showed that the serial exhaustive extraction approach used in this study allowed capturing the antimicrobial and antioxidant metabolites beyond the single extraction, indicating the need for a rigorous choice of an appropriate solvent and method for extracting *P. stemaria*. Further investigation is needed to characterize the active ingredients present in the promising extracts.

## 1. Introduction

Microbial infections are increasing worldwide with antimicrobial resistance emerging as one of the principal public health problems, threatening effective prevention and treatment [1]. Currently, bacterial infections due to antimicrobial resistance claim a minimum of 700 000 lives per year worldwide, including 230,000 people who die from multidrug-resistant tuberculosis. According to estimates, it is projected that by 2050, these infections will cause the deaths of 10 million people per year and will cost a staggering amount of US$100 trillion to the global economy through loss of productivity [2, 3]. Today, the major challenge in global health care is the need for novel, effective, and affordable medicines, especially in developing countries where these infections are more prevalent [4]. Forced with the growing resistance of microbial strains to antibiotics and other drugs, the search for alternative treatment is needed.

Traditionally, the crude extracts of different parts of medicinal plants, including root, stem, flower, fruit, and twigs, are widely used to treat various human diseases [5]. Herbal remedies are also widely embraced in many developed countries, with complementary and alternative medicines (CAM) becoming the mainstream [6]. Moreover, CAM prevention and treatment strategies are reported to have the potential to contribute to the reduction of antibiotic use and, therefore, alleviate the development of antimicrobial resistance [7]. Indeed, one survey of the World Health Organization reports that more than 80% of the world's population still depends upon traditional medicines for various diseases [8]. Medicinal plants have the ability to produce a wide variety of secondary metabolites, like alkaloids, glycosides, terpenoids, saponins, steroids, flavonoids, tannins, quinones, and coumarins, which possess multiple biological activities and can be harnessed for the development of new treatment against microbial infections [9].

*Platycerium stemaria* is an epiphytic medicinal plant of the Polypodiaceae family used to treat many ailments including cardiac palpitations, pulmonary troubles, liver diseases, genital stimulants/depressants, asthma, and infectious diseases [10, 11]. To the best of our knowledge, the biological activities of metabolites from this plant are still to be documented. However, plants belonging to this genus were reported for their antimicrobial activity [12]. Therefore, this work attempted to apply the serial exhaustive extraction method and liquid-liquid partition to investigate the antimicrobial and antioxidant activities of extracts and fractions from *Platycerium stemaria*. Notably, given the gap in scientific knowledge of this medicinal plant, this study was aimed at providing a reasonable starting point for its further investigation as a potential source of active metabolites against microbial pathogens.

## 2. Methodology

### 2.1. Plant Collection

**Figure 1:**
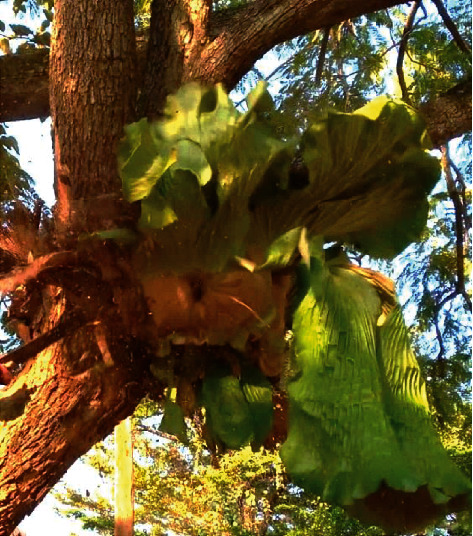
*Platycerium stemaria* (Beauv) Desv. epiphyte of a tree of *Terminalia mantaly*, Yaoundé, Cameroon (July 2, 2020).

The whole plant of *Platycerium stemaria* (Beauv) Desv., growing on the branches of a tree of *Terminalia mantaly* (Figure 1), was harvested in Yaoundé, Cameroon (latitude 3° 42′ N, longitude 11° 20′ E) on July 2, 2020. Mr. Nana Victor made the botanical identification at the Cameroon National Herbarium in Yaoundé, where a voucher specimen was deposited under the reference number 34966 HNC. No permission was necessary for sample collection.

### 2.2. Extracts Preparation Using Serial Exhaustive Extraction Method

**Figure 2:**
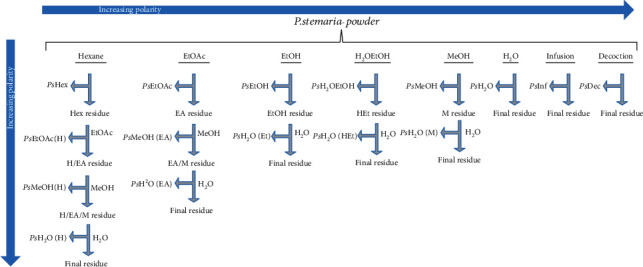
Scheme showing the serial exhaustive extraction method with solvents of increasing polarity from nonpolar (*n*-hexane) to more polar solvent (water) to extract metabolites from the powder of *P. stemaria*.

The fresh plant materials were washed separately with fresh water to remove dirt and other contaminants, shade-dried for three weeks still constant weight, and ground to fine powders. For extraction, 100 g of powder was transferred in different Erlenmeyer flasks and macerated under shaking for 72 hours in hexane, ethyl acetate, ethanol, methanol, hydroethanol (30/70), and distilled water. Infusion and decoction were also prepared. The residues obtained after each of the extraction were submitted to gradual extraction using solvents of increased polarity. The residue from hexane extraction was extracted successively with ethyl acetate, methanol, and water, the residue from the ethyl acetate extraction was extracted with methanol and water, while residues from methanol, ethanol, and hydroethanol were extracted only with water (Figure 2).

The solutions were sieved using hydrophilic cotton and filtered using Whatman filter paper No. 1 after which the organic filtrates were concentrated using a rotary evaporator (BUCHI 461) at 65°C (for hydro-ethanol), at 60°C (for methanol, ethanol) and 40°C (for hexane, ethyl acetate) under reduced pressure till dryness to obtain the crude extract. The aqueous infusion and decoction were evaporated by constant ventilation at 25°C. The extracts were preserved in sterile bottles and conserved at 4°C for further experiments.

### 2.3. Fractionation of Active Extracts Using Liquid-Liquid Partition

The crude extracts of *Ps*H2O (H), *Ps*Hex, *Ps*MeOH (EA), and *Ps*MeOH were selected based on their antibacterial activity and were submitted to fractionation using liquid-liquid partition. Briefly, 10 g of *Ps*H2O (H) extract was dissolved in 50 mL of ethyl acetate and 50 mL of hexane to yield two fractions [*fr*EA(*Ps*H_2_O(H)) and *fr*H(*Ps*H_2_O(H))]; 10 g of *Ps*Hex extract was dissolved in 50 mL hexane and extracted with 50 mL methanol to yield *fr*M(*Ps*Hex) and *fr*H*(Ps*Hex). 10 g of *Ps*MeOH (EA) and *Ps*MeOH was dissolved in 50 mL methanol and extracted with 50 mL hexane to yield six fractions including *fr*H(*Ps*MeOH(EA)), *fr*M(*Ps*MeOH(EA)), and P(*Ps*MeOH (EA)) from extract *Ps*MeOH(EA) and *fr*M(*Ps*MeOH), *fr*H(*Ps*MeOH), and P(*Ps*MeOH) from extract *Ps*MeOH. The afforded fractions were then concentrated using a rotary evaporator. The dried fractions were weighed and the yields calculated relative to the starting crude extracts' weight. Fractions were dissolved in 100% DMSO and progressed for antimicrobial screening.

### 2.4. *In Vitro* Antimicrobial Assay

#### 2.4.1. Microbial Species and Culture Media

Three bacteria, including *Shigella flexineri* NR-518 (SF), *Staphylococcus aureus* ATCC 43300 (SA), and *Klebsiella pneumoniae* ATCC 700603 (KP), and three yeasts (*Candida albicans* NR 29445 (CA1), *Candida albicans* NR 29451 (CA2), and *Candida albicans* NR 2944 (CA3)) were used. The microbial strains were obtained from BEI resources and the American Type Culture Collection. The microorganisms were maintained on an agar slope at 4°C and subcultured before the experiment for 24 h and 48 h, respectively, for bacteria and yeasts.

#### 2.4.2. Preliminary Screening and MIC Determination

The sixteen extracts were screened at 500 *μ*g/mL for their ability to inhibit bacteria/Candida species. Briefly, ninety-two microliters (92 *μ*L) of Muller Hinton broth (MHB) were aseptically introduced into the wells of a 96-well microplate. Eight microliters (8 *μ*L) of each extract, initially prepared at 12.5 mg/mL, were added in wells followed by 100 *μ*L of standardised bacterial suspension (10^6^ CFU/mL) to obtain final volumes of 200 *μ*L. The tests were performed simultaneously for negative control (MHB+pathogens) and sterility control (MHB alone). Ciprofloxacin was used as the positive control and tested at 5 *μ*g/mL. The test was performed in duplicate, and the plates were incubated at 37°C for 24 and 48 hours, respectively, for bacteria and yeasts. The active extracts were further submitted to the dose-response study for MIC determination.

The minimum inhibitory concentration (MIC) of selected extracts and fractions was determined according to the M07-A9 Clinical Laboratory Standards Institute microdilution method using 96-wells microtitre plates. Briefly, 4 *μ*L of extracts and reference drug (Ciprofloxacin) from stock solutions were introduced in the well, followed by the addition of 96 *μ*L of bacteria inoculum standardised at 10^6^ CFU/mL. A blank column was included for sterility control, while bacterial/yeast strains in culture medium without any inhibiting substance were negative control. The concentrations of extracts ranged from 3.905 *μ*g/mL to 500 *μ*g/mL, and that of Ciprofloxacin ranged from 0.562 *μ*g/mL to 128 *μ*g/mL. After 24 hours of incubation at 37°C, the turbidity was observed as an indication of growth. MIC was defined as the lowest concentration inhibiting the visible growth of microorganisms. All tests were performed in duplicate [13].

### 2.5. *In Vitro* Antioxidant Assay

#### 2.5.1. DPPH Radical Scavenging Assay

The free radical scavenging assay was performed to study the antioxidant potency of extracts, based on the scavenging activity of 1, 1-diphenyl-2- picrylhydrazyl (DPPH) using the method described by Stratil et al. [14]. A sample (3 mL) was mixed with a DPPH solution (Sigma) in HPLC grade methanol (Merck), vortexed at room temperature, and left standing for 10 min. The UV/VIS absorbance was measured at *λ* = 517 nm serving the methanol without DPPH solution as the blank solution. A reference solution of vitamin C (Sigma Aldrich) in methanol was used, taking 100% radical scavenging activity. The concentration of extracts ranged from 500, 250, 125, 62.5, 31.25, 15.625, and 7.812 *μ*g/mL. The assay was performed in duplicate and two times. The scavenging percentage was calculated with the following equation:

(1) $$\begin{array}{c} \%\text{radical scavenging activity}=\left(\text{A}0-\text{A}5\right)\times 100/\text{A}0. \end{array}$$

(Where A0 and A5 are the absorbance values of DPPH+Sample solution at 0.0 min and after 0.5 min, respectively).

The % radical scavenging activity was used to determine the EC_50_ and the Antiradical power (ARP), which is the inverse of the EC_50_.

#### 2.5.2. Ferric Reducing Antioxidant Power Assay

The capacity of each plant extracts to reduce the ferric-ferry cyanide complex to the ferrous-ferry cyanide complex was determined as described by Stratil et al. [14] with slight modifications. Briefly, 2.5 mL of different plant extract solutions were mixed with 2.5 mL of phosphate buffer (pH 6.6) and 2.5 mL of 1% potassium ferry cyanide. The mixture was incubated at 50°C for 20 min. After incubation, 2.5 mL of 10% TCA was added to the mixture and centrifuged at 10000 rpm for 10 min. 2.5 mL of supernatant was mixed with 2.5 mL of distilled water and 0.5 mL of 0.1% ferric chloride. Then, the absorbance was measured at 510 nm by using a UV spectrophotometer. An increment of the absorbance of the reaction mixture indicates increased reducing power. A similar method was adopted for Ascorbic acid used as a positive control. All the tests were performed in duplicates. From a concentration-activity curve of vitamin C used as standard, the optical densities of the test wells were projected, and the results expressed as *μ*g equivalent Vit C/g of extracts.

### 2.6. Phytochemical Screening

The presence of major phytochemical classes of compounds such as alkaloids, anthraquinones, flavonoids, glycosides, phenols, saponins, sterols, tannins, and triterpenoids were qualitatively evaluated in the plant extracts according to previously described methods [13].

### 2.7. Statistical Analyses

The data were subjected to one-way analysis of variance (ANOVA) using the Statistical Package for the Social Sciences (SPSS, version 17.0) program. Microsoft Excel 2016 software for Windows was used to calculate, IC_50_, EC_50_, ARP, and plot the standard curve for vitamin C. The results were presented as the mean ± SD.

## 3. Results

### 3.1. *In Vitro* Antimicrobial Potential of the Extracts

#### 3.1.1. Extraction Yield and Antimicrobial Activity of Extracts

The serial exhaustive extraction system from the nonpolar to the polar solvents yielded sixteen extracts with the yield values ranging from 1.484% to 6.002%, varying as a function of extraction solvent (Table 1). In the first extraction, ethanol followed by water exhibited better extraction yields *Ps*EtOH (6.002%) and *Ps*H_2_O (4.32%), respectively, contrary to decoction and ethyl acetate showing weak yields as noticed with the extraction of 1.66% and 1.62% for extracts *Ps*Dec and *Ps*EtOAc, respectively. The gradual extraction of the residue of *P. stemaria* with more polar solvents produced extracts with the highest extraction yield. For instance, the yield of *Ps*H_2_O(H) (3.478%) was 1.58, 1.44, and 1.55 time higher as compared to *Ps*MeOH(H), *Ps*EtOAc(H), and *Ps*Hex, respectively.

The results of the antimicrobial screening at 500 *μ*g/mL showed that although none of the extracts inhibited *Candida albicans* strains, four (25%) extracts, including *Ps*Hex, *Ps*H_2_O(H), *Ps*MeOH(EA), and *Ps*MeOH exhibited antibacterial activity in a strain-specific manner. *Ps*Hex and *Ps*MeOH were active on *S. flexineri*, while *Ps*MeOH(EA) and *Ps*H_2_O(H) inhibited, respectively, *S. aureus* and *K. pneumonia*. The four extracts were progressed to a dose-response study for MIC determination. Extract *Ps*H_2_O(H) was active against *K. pneumonia* with MIC of 31.25 *μ*g/mL, while *Ps*MeOH(EA) inhibited *S. aureus* with MIC of 250 *μ*g/mL, and *Ps*Hex and *Ps*MeOH inhibited *S. flexineri* with MIC values of 500 *μ*g/mL.

Overall, the results revealed the effect of the serial exhaustive system on the activity of extracts. Indeed, while *Ps*Hex extract was active, none of the extracts (*Ps*MeOH(H), *Ps*EtOAc(H), and *Ps*H_2_O(H)) obtained from the extraction of the initial residue displayed activity. In the contrary, the *Ps*MeOH (EA) extract obtained from the extraction of the residue from *Ps*EtOAc was active, while the initial ethyl acetate extract (*Ps*EtOAc) and extract *Ps*H2O(EA) (extracted from the residue of *Ps*MeOH(EA)) were inactive. Finally, methanol extract *Ps*MeOH was active, while *Ps*H_2_O(M) extracted from *Ps*MeOH residue was inactive. This analysis reveals that metabolites with antimicrobial activity are concentrated in a specific fraction in *P. stemaria* and, therefore, justifying the serial exhaustive extraction strategy applied to reveal their potency.

#### 3.1.2. Activity of Fractions against Bacteria Pathogens

The selected extracts were later subjected to liquid-liquid partition with the aim to further concentrate the active metabolites. The ten fractions obtained were submitted to the minimum inhibitory concentration (MIC) determination as presented in Table 2.

The fractionation of *Ps*Hex extract yielded the fractions *fr*H(*Ps*Hex) and *fr*M(*Ps*Hex) with extraction yield of 1.7 and 0.54%, respectively, both inactive against *S. flexineri*. The *Ps*MeOH extract yielded three fractions *fr*M (*Ps*MeOH), *fr*H (*Ps*MeOH), and P(*Ps*MeOH) with the yield of 0.9, 0.4, and 0.160%, respectively. Only P(*Ps*MeOH) was active on *S. flexineri* (MIC of 500 *μ*g/mL). Similarly, the fractionation of *Ps*MeOH(EA) also yielded three fractions among which only *fr*M(*Ps*MeOH(EA)) with the yield of 2.4% was active on *S. aureus* (MIC of 500 *μ*g/mL). Finally, extract *Ps*H_2_O(H) was separated into two fractions of 1.01% (*fr*H(*Ps*H_2_O(H))) and 2.4% (*fr*EA(*Ps*H_2_O(H))) both active on *K. pneumonia* with the MIC value of 500 *μ*g/mL.

In general, the liquid-liquid fractionation of active extracts led to a substantial decrease and loss of potency. In fact, while none of the fractions from *Ps*Hex were active, fractions from *Ps*MeOH(EA) displayed a two-fold decrease in potency, and fractions from *Ps*H_2_O(H) displayed 16-fold decrease in potency. This loss and decrease of the activity of these fractions could be related to the loss of synergistic interaction (due to separation) existing among compounds present in the mixture.

### 3.2. *In Vitro* Antioxidant Potentials of the Extracts

The fractions having loss their antimicrobial potency, the four initial active extracts were submitted to the evaluation of their antioxidant activity using both DPPH and FRAP assays.

#### 3.2.1. DPPH Radical Scavenging Activity of Extracts

The DPPH radical scavenging activity of extracts is presented in Table 3. The quantitative analysis of the scavenging ability showed that two extracts exhibited very good free radical scavenging activities, while the two others portrayed no activity even at the maximum concentration of 500 *μ*g/mL.

Overall, the IC_50_ values ranged from 41.69 to >500 *μ*g/mL. Extract *Ps*MeOH(EA) exhibited the highest DPPH radical scavenging activity with IC_50_ of 41.69 *μ*g/mL as compared to *Ps*MeOH extract (IC_50_ of 51.35 *μ*g/mL). Both extracts displayed an antiradical power of 47.96 × 10^−5^ and 38.94 × 10^−5^. Indeed, the antiradical power measures antiradical effectiveness; the higher this value, the more the extract has a strong proton donor capacity to stabilize the radical. However, both extracts were less active than the ascorbic acid used as the positive control (IC_50_ 8.92 *μ*g/mL; ARP 224.21 × 10^−5^).

#### 3.2.2. Ferric Ion Reducing Antioxidant Power (FRAP) of Extracts

FRAP assay showed that there was a correlation between the concentration of the extracts and their reducing power. From the data, the absorbance of the Fe^2+^-extract complex increased with the concentration of extract, and this absorbance is proportional to the amount of Fe^3+^ ions reduced. Thus, a high absorbance would reflect a large amount of Fe^2+^-extract complex and, therefore, an intense reducing activity of these extracts. Using the standard curve from vitamin C, the absorbances were used to quantify the Fe^3+^ reducing power of different plant extracts.

Table 4 shows that all the extracts tested displayed a certain degree of inhibition; however, extracts *Ps*Hex (0.54-3.48 *μ*g equivalent Vit C/g of extract) and *Ps*H_2_O(H) (2.49-13.77 *μ*g equivalent Vit C/g of extract) were poorly active. On the contrary, at 250 *μ*g/mL the activity extract *Ps*MeOH(EA) was significantly (*P* ≤ 0.05) higher than those of the *Ps*MeOH. However, at the maximum concentration of 500 *μ*g/mL, the reducing capacities of both extracts of 58.99 *μ*g equivalent Vit C/g of extract (*Ps*MeOH) and 61.53 *μ*g equivalent Vit C/g of extract (*Ps*MeOH(EA)) were not significantly different (*P* > 0.05). In general, the results from both the DPPH and FRAP assays showed that *Ps*MeOH and *Ps*MeOH(EA) extracts presented greater antioxidant potentials. The high antioxidant effect of these methanolic extracts could be due to the diversity of compounds extracted.

## 4. Phytochemical Composition of Extracts

The selected extracts were submitted to qualitative phytochemical analysis, and the results are summarized in Table 4.

The phytochemical screening presented in Table 5 shows that the families of secondary metabolites involved in the biological activities are unevenly distributed in the different plant extracts. Indeed, although entirely different in chemical composition, all the extracts contain phenols and tannins. Extracts *Ps*MeOH(EA) and *Ps*MeOH with, respectively, 7 and 6 groups of metabolites showed a great abundance in phytochemicals. Extract *Ps*Hex was the poorest, with only two classes identified. In general, this test revealed the presence of flavonoids, glycosides, phenols, tannins, terpenoids, saponins, and anthraquinones. The results also show that alkaloids and sterols were absent in all extracts.

## 5. Discussion

Today, antibiotic-resistant pathogens are a severe threat to public health. This problem becomes even worse with a decline in the supply of new antibiotics [15]. For olden times, medicinal plants were used to heal human diseases, and stand as part of human history. Moreover, the history of modern medicine is full of remarkable stories of how the exploration of medicinal plants led to discovering natural products that profoundly impacted advances in biology and inspired drug discovery and therapy [16]. The discovery of new potential antibiotics could benefit from the exploration of underinvestigated medicinal plants. Therefore, in this study, the antimicrobial potential of extracts and fractions from *Platycerium stemaria* used to treat various human diseases in African traditional medicines was explored. More specifically, this study was designed to identify the appropriate solvent system to concentrate antimicrobial and antioxidant metabolites present in *P. stemaria* as an attempt to discover new antibiotics.

The results showed that the serial exhaustive extraction method involving the successive extraction with solvents of increasing polarity from *n*-hexane to water to ensure the extraction of a wide range of phytochemicals with different polarities have significantly impacted the extraction yields and activity. This method was reported as suitable to improve the extraction of metabolites from various plant materials [17, 18]. Moreover, it is well-known that depending on their chemical nature, various phytochemicals are extracted in solvents of different polarities as no single solvent may be reliable to extract all the phytochemicals [19]. This methodology is particularly useful while investigating medicinal plants with no existing previous study. Thus, given the gap in knowledge regarding the biological and phytochemical investigation of *P. stemaria*, the serial exhaustive extraction is appropriate for an initial investigation.

The sixteen crude extracts obtained were submitted to antimicrobial screening to identify only four extracts exhibiting selective antibacterial activity against Gram-negative bacteria *S. flexineri* (*Ps*Hex and *Ps*MeOH) and *K. pneumonia* (*Ps*H2O(H)) and Gram-positive bacteria *S. aureus* (*Ps*MeOH(EA)). This differential activity profile of extracts could be related to the mechanism of action of their active ingredients. More investigation into these metabolites and their possible mechanism of action could provide insightful informations. Indeed, the phytochemical analysis of promising extracts revealed a differential composition in terms of metabolite classes, including flavonoids, glycosides, phenols, tannins, terpenoids, saponins, and anthraquinones. Of note, phenols and tannins were present in all four extracts. Metabolites belonging to these classes were reported for their antibacterial activity [20–23]. The 4 selected extracts were fractionated using liquid-liquid partition to concentrate potential active ingredients into more simplified fractions. However, the antimicrobial testing of the afforded fractions against the three sensitive bacteria revealed an important decrease in the potency. Fractions from extract *Ps*Hex were inactive up to the concentration of 500 *μ*g/mL, while fractions from *Ps*MeOH(EA) and *Ps*H_2_O(H) with MICs of 500 *μ*g/mL displayed a 2 and 16 fold decrease in potency, respectively. The loss of potency observed likely suggests that the activity of parent extracts could be associated with synergistic interactions between two or several metabolites present in each of these crude extracts. Indeed, medicinal plants contain myriads of compounds that act together to exhibit a specific function [24, 25]. The activity of fractions having decreased, we opted to explore the antioxidant activity of the four initial parent extracts. The results showed that in both DPPH and FRAP assays, extracts *Ps*Hex and *Ps*H_2_O(H) were inactive up to 500 *μ*g/mL while *Ps*MeOH and *Ps*MeOH(EA) exhibited potency. The observed potency corroborates the presence of various groups of antioxidant metabolites in the active extracts (*Ps*MeOH and *Ps*MeOH(EA)). Future metabolomics-guided investigation of these two active extracts could warrant isolation and characterization of promising metabolites for further development.

## 6. Conclusion

This study used a serial exhaustive extraction approach to investigate the antimicrobial and antioxidant potentials of extracts from *Platycerium stemaria*. The investigation revealed a significant variation in extraction yield and antimicrobial activity of extracts as a function of solvent and microbial pathogen used. Moreover, among the four antimicrobial extracts identified, two extracts exhibited both radical scavenging and ferric reducing antioxidant power. These extracts also contain several antimicrobial and antioxidants metabolites identified by phytochemical screening, suggesting the need for further investigation to characterize the observed activities. Overall, this pioneering study is of particular importance in the attempt to fill the gap in phytochemical and pharmacological knowledge of *P. stemaria*. It showed that serial exhaustive extraction approach led to active metabolites being located beyond the single extraction, indicating that selecting the appropriate solvent and the method for extraction is very much essential to locate antimicrobial metabolites in *P. stemaria*. Further investigation is needed to characterize the active ingredients present in the promising extracts identified in this study.

## Figures and Tables

**Table 1:** Yield (%) and minimum inhibitory (MIC) concentrations (*μ*g/mL) of extracts of *Platycerium stemaria*.

Extracts	Yield (%)	SF	SA	KP	CA1	CA2	CA3
*Ps*Hex	2.242	500 ± 0.00	—	—	—	—	—
*Ps*EtOAc(H)	2.414	—	—	—	—	—	—
*Ps*MeOH(H)	2.202	—	—	—	—	—	—
*Ps*H_2_O(H)	3.478	—	—	31.25 ± 0.00	—	—	—
*Ps*EtOAc	1.62	—	—	—	—	—	—
*Ps*MeOH(EA)	4.32	—	250 ± 0.00	—	—	—	—
*Ps*H_2_O(EA)	2.542	—	—	—	—	—	—
*Ps*EtOH	6.002	—	—	—	—	—	—
*Ps*H_2_O(Et)	1.484	—	—	—	—	—	—
*Ps*H_2_OEtOH	3.298	—	—	—	—	—	—
*Ps*H_2_O(HEt)	3.656	—	—	—	—	—	—
*Ps*MeOH	1.462	500 ± 0.00	—	—	—	—	—
*Ps*H_2_O(M)	2.44	—	—	—	—	—	—
*Ps*H_2_O	4.232	—	—	—	—	—	—
*Ps*Inf	3.1	—	—	—	—	—	—
*Ps*Dec	1.66	—	—	—	—	—	—

CA1: *Candida albicans* NR 29445; CA2: *Candida albicans* NR 29451; CA3: *Candida albicans* NR 29441; KP: *Klebsiella pneumonia*; SF: *Shigella flexineri*; SA: *Staphylococcus aureus*. *Ps*Hex: hexane extract of *Platycerium stemaria*; *Ps*EtOAc(H): ethyl acetate extract from the hexane residue of *P. stemaria*; *Ps*MeOH(H): methanol extract from the hexane/EtOAc residue of *P. stemaria*; *Ps*H_2_O(H): aqueous extract from the hexane/EtOAc/MeOH residue of *P. stemaria*; *Ps*EtOAc: ethyl acetate extract of *P. stemaria*; *Ps*MeOH(EA): methanol extract from the ethyl acetate residue of *P. stemaria*; *Ps*H2O(EA): aqueous extract from the ethyl acetate/MeOH residue of *P. stemaria*; *Ps*EtOH: Ethanol extract of *P. stemaria*; *Ps*H_2_O(Et): aqueous extract from the ethanol residue of *P. stemaria*; *Ps*H_2_OEtOH: hydroethanol extract of *P. stemaria*; *Ps*H_2_O(HEt): aqueous extract from the hydroethanol residue of *P. stemaria*; *Ps*MeOH: methanol extract of the *P. stemaria*; *Ps*H_2_O(M): aqueous extract from the methanol residue of *P. stemaria*; *Ps*H_2_O: aqueous extract of *P. stemaria*; *Ps*Inf: infusion extract of *P. stemaria*; *Ps*Dec: decoction extract of *P. stemaria*.

**Table 2:** Minimum inhibitory (MIC) concentrations (*μ*g/mL) of fractions.

Extracts	Fractions	Yield (%)	SF	SA	KP
*Ps*Hex		2.242	500 ± 0.00	—	—
	*fr*H(*Ps*Hex)	1.70	>500	—	—
	*fr*M(*Ps*Hex)	0.54	>500	—	—

*Ps*H_2_O(H)		3.478	—	—	31.25 ± 0.00
	*fr*H(*Ps*H_2_O(H))	1.01	—	—	500 ± 0.00
	*fr*EA(*Ps*H_2_O(H))	2.4	—	—	500 ± 0.00

*Ps*MeOH(EA)		4.32	—	250 ± 0.00	—
	*fr*H(*Ps*MeOH(EA))	1.0	—	>500	—
	*fr*M(*Ps*MeOH(EA))	2.4	—	500 ± 0.00	—
	P1(*Ps*MeOH (EA))	0.80	—	>500	—

*Ps*MeOH		1.462	500 ± 0.00	—	—
	*fr*M(*Ps*MeOH)	0.9	>500	—	—
	*fr*H(*Ps*MeOH)	0.4	>500	—	—
	P(*Ps*MeOH)	0.160	500 ± 0.00	—	—

KP: *Klebsiella pneumonia*; SF: *Shigella flexineri*; SA: *Staphylococcus aureus*; *Ps*Hex: Hexane extract of *P. stemaria*; *fr*H(*Ps*Hex): hexanic fraction from *Ps*Hex; *fr*M(PsHex): methanol fraction from *Ps*Hex; *Ps*H_2_O(H): aqueous extract from the hexane/EtOAc/MeOH residue of *P. stemaria*; *fr*H(*Ps*H_2_O(H)): hexane fraction from *Ps*H_2_O(H); *fr*EA(*Ps*H_2_O(H)): ethyl acetate fraction from *Ps*H_2_O(H); *Ps*MeOH(EA): methanol extract from the ethyl acetate residue of *P. stemaria*; *fr*H(*Ps*MeOH(EA)): hexane fraction from *Ps*MeOH(EA); *fr*M(*Ps*MeOH(EA)): methanol fraction from *Ps*MeOH(EA); P(*Ps*MeOH (EA)): precipitate fraction from *Ps*MeOH (EA); *Ps*MeOH: methanol extract of *P. stemaria*; *fr*M(*Ps*MeOH): methanolic fraction from *Ps*MeOH; *fr*H(*Ps*MeOH): hexane fraction from *Ps*MeOH; P(*Ps*MeOH): precipitate fraction from *Ps*MeOH.

**Table 3:** 50% Radical scavenging activity (RSA_50_), 50% efficient concentration (EC_50_), and antiradical power against DPPH radical.

Extracts	IC_50_ ± SD (*μ*g/mL)	EC_50_	ARP
*Ps*Hex	>500	NA	NA
*Ps*H_2_O(H)	>500	NA	NA
*Ps*MeOH(EA)	41.69 ± 0.96^a^	0.20848 × 10^4a^	47.96 × 10^−5a^
*Ps*MeOH	51.35 ± 1.23^b^	0.25675 × 10^4a^	38.94 × 10^−5b^
Vitamin C	8.92 ± 1.065^c^	0.0446 × 10^4b^	224.21 × 10^−5c^

ARP: antiradical power; EC_50_: 50% efficient concentration; IC_50_: 50% inhibition Concentration; *Ps*Hex: hexane extract of *P. stemaria*; *Ps*H_2_O(H): aqueous extract from the hexane/EtOAc/MeOH residue of *P. stemaria*; *Ps*EtOAc: ethyl acetate extract of *P. stemaria*; *Ps*MeOH(EA): methanol extract from the ethyl acetate residue of *P. stemaria*; *Ps*MeOH: methanol extract of the *P. stemaria*. a, b, and c are statistically different at *P* ≤ 0.05, same number means no difference and different numbers means significantly different.

**Table 4:** Quantitative evaluation of Fe^3+^ reducing power of different plant extracts.

Extracts concentration (*μ*g/mL)	*μ*g equivalent Vit C/g of extract			
	*Ps*MeOH	*Ps*Hex	*Ps*H_2_O(H)	*Ps*MeOH(EA)
7.812	0.34 ± 0.84^a^	−2.86 ± 0.46^a^	2.49 ± 4.5^a^	1.87 ± 0.36^a^
15.625	1.75 ± 0.39^a^	0.54 ± 0.35^a^	3.8 ± 5.4^a^	4.85 ± 0.43^a^
31.25	3.17 ± 0.38^b^	0.19 ± 0.34^a^	4.18 ± 5.5^ab^	8.18 ± 0.52^b^
62.5	9.01 ± 1.3^c^	0.82 ± 0.48^b^	5.14 ± 4.8^abc^	9.37 ± 0.46^bc^
125	18.25 ± 0.11^d^	1.57 ± 0.76^b^	7.13 ± 4.8^abc^	20.70 ± 0.94^d^
250	30.14 ± 0.001^e^	1.89 ± 1.09^b^	7.80 ± 5.08^c^	40.06 ± 0.39^f^
500	58.99 ± 0.33^g^	3.48 ± 0.42^b^	13.77 ± 6.57^c^	61.53 ± 0.00^g^

*Ps*Hex: hexane extract of *P. stemaria*; *Ps*H_2_O(H): aqueous extract from the hexane/EtOAc/MeOH residue of *P. stemaria*; *Ps*EtOAc: ethyl acetate extract of *P. stemaria*; *Ps*MeOH(EA): methanol extract from the ethyl acetate residue of *P. stemaria*; *Ps*MeOH: methanol extract of the *P. stemaria*. Along and across the line, values carrying the same letters are not significantly different (*P* > 0.05); different letters indicate values are significant at (*P* ≤ 0.05).

**Table 5:** Phytochemical composition of *Platycerium stemaria* extracts.

Phytochemicals	*Ps*MeOH	*Ps*Hex	*Ps*H_2_O(H)	*Ps*MeOH(EA)
Alkaloids	-	-	-	-
Flavonoids	**+**	-	**+**	**+**
Glycosides	-	-	-	**+**
Phenols	**+**	**+**	**+**	**+**
Anthraquinones	**+**	-	**+**	**+**
Tannins	**+**	**+**	**+**	**+**
Triterpenoids	**+**	-	**+**	**+**
Saponins	**+**	-	-	**+**
Sterols	-	-	-	-

*Ps*Hex: hexane extract of *P. stemaria*; *Ps*H_2_O(H): aqueous extract from the hexane/EtOAc/MeOH residue of *P. stemaria*; *Ps*EtOAc: ethyl acetate extract of *P. stemaria*; *Ps*MeOH(EA): methanol extract from the ethyl acetate residue of *P. stemaria*; *Ps*MeOH: methanol extract of the *P. stemaria*; *+*: presence; -: absence.

## Data Availability

Data used to support the findings of this study are included within the article.

## Abbreviations

ARP: Antiradical power
EC_50_: 50% efficient concentration
IC_50_: 50% inhibition concentration
CA1: *Candida albicans* NR 29445
CA2: *Candida albicans* NR 29451
CA3: *Candida albicans* NR 29441
DPPH: 1, 1-diphenyl-2- picrylhydrazyl
KP: *Klebsiella pneumonia*
MIC: Minimum inhibitory concentration
*Ps*Hex: Hexane extract of *Platycerium stemaria*
*Ps*EtOAc(H): Ethyl acetate extract from the hexane residue of *P. stemaria*
*Ps*MeOH(H): Methanol extract from the hexane/EtOAc residue of *P. stemaria*
*Ps*H_2_O(H): Aqueous extract from the hexane/EtOAc/MeOH residue of *P. stemaria*
*Ps*EtOAc: Ethyl acetate extract of *P. stemaria*
*Ps*MeOH(EA): Methanol extract from the ethyl acetate residue of *P. stemaria*
*Ps*H2O(EA): Aqueous extract from the ethyl acetate/MeOH residue of *P. stemaria*
*Ps*EtOH: Ethanol extract of *P. stemaria*
*Ps*H_2_O(Et): Aqueous extract from the ethanol residue of *P. stemaria*
*Ps*H_2_OEtOH: Hydroethanol extract of *P. stemaria*
*Ps*H_2_O(HEt): Aqueous extract from the hydroethanol residue of *P. stemaria*
*Ps*MeOH: Methanol extract of the *P. stemaria*
*Ps*H_2_O(M): Aqueous extract from the methanol residue of *P. stemaria*
*Ps*H_2_O: Aqueous extract of *P. stemaria*
*Ps*Inf: Infusion extract of *P. stemaria*
*Ps*Dec: Decoction extract of *P. stemaria*
SF: *Shigella flexineri*
SA: *Staphylococcus aureus*
*fr*H(*Ps*Hex): Hexanic fraction from *Ps*Hex
*fr*M(PsHex): Methanol fraction from *Ps*Hex
*fr*H(*Ps*H_2_O(H)): Hexane fraction from *Ps*H_2_O(H)
*fr*EA(*Ps*H_2_O(H)): Ethyl acetate fraction from *Ps*H_2_O(H)
*fr*H(*Ps*MeOH(EA)): Hexane fraction from *Ps*MeOH(EA)
*fr*M(*Ps*MeOH(EA)): Methanol fraction from *Ps*MeOH(EA)
P(*Ps*MeOH (EA)): Precipitate fraction from *Ps*MeOH (EA)
*fr*M(*Ps*MeOH): Methanolic fraction from *Ps*MeOH
*fr*H(*Ps*MeOH): Hexane fraction from *Ps*MeOH
P(*Ps*MeOH): Precipitate fraction from *Ps*MeOH.
